# The effect of web quest and team-based learning on students’ self-regulation

**Published:** 2016-04

**Authors:** ZOHREH BADIYEPEYMAIE JAHROMI, LEILI MOSALANEJAD, RITA REZAEE

**Affiliations:** 1 Nursing Department, Jahrom University of Medical Sciences, Jahrom, Iran;; 2 Medical Education Center, Jahrom University of Medical Sciences, Jahrom, Iran;; 3 Quality Improvement in Clinical Education Research Center, Education Development Center, Shiraz University of Medical Sciences, Shiraz, Iran

**Keywords:** Clinical, Nursing, Training, Students, Learning, Internet, Problem-based learning

## Abstract

**Introduction:**

In this study, the authors aimed to examine the effects of cooperative learning methods using Web Quest and team-based learning on students’ self-direction, self-regulation, and academic achievement.

**Method:**

This is a comparative study of students taking a course in mental health and psychiatric disorders. In two consecutive years, a group of students were trained using the WebQuest approach as a teaching strategy (n = 38), while the other group was taught using team-based learning (n=39). Data gathering was based on Guglielmino’s self-directed learning readiness scale (SDLRS) and Buford’s self-regulation questionnaire. The data were analyzed by descriptive test using M (IQR), Wilcoxon signed-rank test, and the Mann–Whitney U-test in SPSS software, version 13. p<0.05 was considered as the significance level.

**Results:**

The results of the Mann–Whitney U test showed that the participants’ self- directed (self-management) and self-regulated learning differed between the two groups (p=0.04 and p=0.01, respectively). Wilcoxon test revealed that self-directed learning indices (self-control and self-management) were differed between the two strategies before and after the intervention. However, the scores related to learning (students’ final scores) were higher in the WebQuest approach than in team-based learning.

**Conclusion:**

By employing modern educational approaches, students are not only more successful in their studies but also acquire the necessary professional skills for future performance. Further research to compare the effects of new methods of teaching is required.

## Introduction

Information explosion is a constant challenge to education, but it must lead to empowerment; individuals need to acquire the capacity to make the right decisions and implement them effectively. The objective of most educational courses is to communicate information, encourage higher-order thinking, and develop the students’ problem-solving skills (1, 2).

With the development of E-learning in this technologically advanced age, traditional teaching methods have changed drastically (3). Accordingly, the National League for Nursing (NLN) encourages the use of modern technology in nursing education (4). Graduates need to adapt to ever-increasing medical knowledge, new developments in clinical environments, and rapid advancement in technology and the consequent changes in the society’s academic needs (5, 6).

WebQuest is a teaching technique used to impart certain subjects with special needs. It is a learner-centered strategy which involves the students in the development of knowledge and the creation of new concepts (7). It is not a tool to facilitate memorization of information, but rather, a method to encourage creativity, development, inquiry, analysis, reflection, synthesis, and evaluation (8). To use WebQuest, it is necessary for teachers to find helpful websites, create harmony between the learner and the sources, and encourage the learner to think, use the media and fulfill the high expectations set for them (9-16).

Nowadays, integrated learning is considered a very helpful tool in education at medical universities. Similarly, team-based learning (TBL) is an effective educational method in which the three components of student, teacher, and curriculum should be perfectly integrated (16-18).

TBL can be employed in large classes. It consists of a sequential three-stage structure: 1) Readiness: the students study the lesson already assigned by the teacher; 2) Readiness assurance test: the students demonstrate their readiness through individual and team assurance tests; and 3) Application: the students use the learned concepts to solve problems designed by the faculty and analyze them as a team (19). Among the benefits of TBL, as cited in various studies, are students’ higher involvement in learning, higher-quality communication, and better exam grades (17).

A combination of TBL and WebQuest as an educational technique can, through the resultant synergy, positively affect the students’ learning and help them make the most of their education(17).

One of the most important stages in student maturation is the recognition and acceptance of self-direction. In view of its various benefits, self-directed learning is highly emphasized in educational and organizational environments and deemed as a necessary skill to learning and working in the 21st century (20). As one of the major components of problem-solving skills, self-directed learning is essential to the clinical competence of medical science graduates. The teachers’ awareness about the students’ self-directed learning levels will enable them to adapt their educational programs to the students’ readiness for self-directed learning and select the best teaching strategies(21).

Many studies have addressed TBL, the factors that affect one’s problem-solving skills and academic success, and self-directed learning. However, there are few studies that investigate the effects of integrated learning strategies and their cognitive and behavioral consequences. In many developing countries, including Iran, active teaching and learning methods are new, and despite the efforts to employ such methods, any type of reform in education has proved difficult in the face of the conventional and passive approaches. On the other hand, the educational as well as cognitive superiority of the new methods over the conventional approaches remains debatable, and there is room for further investigation(22, 23).

Psychiatric issues, particularly the symptoms and ways to treat patients, are among the most difficult subjects to teach. Hence, it is necessary to use methods that will help to provide the students with a better understanding of the problems and encourage them to analyze the issues. 

The purpose of this study was to determine the effects of cooperative learning methods using WebQuest and TBL approaches on the students’ self-direction, self-regulation and academic achievement. 

## Methods

In this experimental and comparative study, nursing students at Jahrom University of Medical Sciences in Iran were taught using Web Quest as a student-centered educational strategy and TBL over two sequential years from 2013 to 2014. These participants, who were divided into two groups, were taking a course in mental health and psychiatric disorders. 

In the first group, the students were placed in four teams of 10 each according to their student I.D. numbers, using WebQuest. Each team, which were given a name chosen by its members, presented its work, and the results, which included an examination of the scenarios and the case studies, were graded. The students were told that this would not influence their actual final grades, but active participation in the group activities would be graded and recorded as a quiz grade. All students were taught the general concepts of the course in the odd sessions, while in the even sessions, they were taught the techniques to treat the disorders, nursing management, and the manner of communicating with patients. At the end of the odd sessions, the students answered four multiple-choice questions as a team. The questions were intended as individual and group assurance tests. In the even sessions, the students watched 15-minute long videos related to the disorders in the study that were brought in by each team. Subsequently, four study cases of the disorder were discussed briefly and the teams was then given an hour to answer the questions about the techniques related to patient communication, nursing management, and other psychosocial approaches during therapy. 

Students were also tasked to learn about treatments and drugs used. Even though drug therapy for each disorder was not a necessary part of the theoretical course and was a part of the internship, it was felt that it was helpful for students to learn it at this time. Because of time limitations, drug therapy related to each disorder was limited to drug classification. This program would last for 15 minutes at the most. 

Overall, the students’ activity in this first group involved finding the best approach, sourcing for videos related to the disorders discussed, and finding a list of commonly administered medicines. Utilizing the Internet as their main research resource, the students used smart phones, tablets, and laptops in class and had access to a nearby Internet cafe at certain times.

In the second group, the students were trained using TBL as a teaching strategy. These students had been trained in the previous year. In this group, the teacher would impart, the concepts, and in the following session, the teacher raised a few challenging questions concerning communication with patients and psychosocial therapy. These questions had no definite answers as they aimed at challenging the students to find various answers. Once divided into several teams of 8–10 each, the students were given a scenario about a certain aspect of the disorder being studied, and had 30–45 minutes to discuss it within their teams. Subsequently, the students shared the results of their discussions with the class. 

Two separate groups were selected for this paper’s study so that a larger number of acceptable samples could be made available; the effects of the approaches did not interrupt each other. Each approach was established, and the results were tangible. The teaching plan was as follows: planning of a problem or scenario, working collectively to obtain a response, intra-group discussion, having each group present their responses, and the teacher’s comments on the discussed issues.

Two questionnaires were used for the participants: Guglielmino’s self-directed learning readiness scale (SDLRS) and Buford’s self-regulation questionnaire. (SDLRS) is a self-report 41-item questionnaire with five parts based on a Likert scale ranging from “rarely” (1) to “always”(5), and it consists of three sections: self-management, learning engagement, and self-control. The internal consistency of the questions was 0.95, and the reliability of the re-test was 0.68. Each section was scored out of 100: scores below 33.3 were considered as low, between 33.3 and 66.7 as medium, and above 66.7 as high. The Cronbach’s alpha of the sub-scales of self-management, learning tendency, and self-control were 0.81, 0.78, and 0.84, respectively. In 2012, Nadi and Sadjadian used this questionnaire on 1135 medicine and dentistry students, and the reliability and validity of the test were confirmed. The maximum and minimum scores of this tool were 205 and 41, respectively (24-26).

The 14-item self-regulation questionnaire was developed by Buford et al, and standardized by Kadivar in 2001. The total validity coefficient of the questionnaire based on Cronbach’s alpha was calculated to be 0.71. The validities of the sub-scales of cognitive and metacognitive strategies were 0.70 and 0.68, respectively. Regarding the structure, the factor results showed that the correlation coefficient of the questions was acceptable and the evaluation tool consisted of two factors. The value of the factors was acceptable and the tool could determine 0.52 of the self-report variance. The structural validity was satisfactory. There were five possible answers for each question: “I totally agree,” “I agree,” “I’m not sure,” “I disagree,” and “I totally disagree.” Each question was scored from 1 to 5, except for questions 5, 13, and 14, which were scored in the reverse (27, 28).

The instruments used in this study consisted of descriptive test as a median and interquartile range [Mean (IQR)]. The data were analyzed using Wilcoxon signed-rank test, the Mann–Whitney U-test and Student t-test as statistical tests in SPSS software version 13. p<0.05 was considered as the level of significance. 

## Results

The Kolmogorov–Smirnov test showed that there was no normality in some indices; therefore, a nonparametric test was used for data analysis. The median and interquartile range or M(IQR) of indices are shown in Table 1. The WebQuest group showed higher levels of self-control, self-management, and self-regulation after educational intervention. In the TBL group, the self-control median range was higher than before the intervention, but the median range for self-regulated learning and self-management had decreased. There was no change in the level of self-engagement for both groups (Table 1).

**Table1:** Descriptive analysis from self-directed learning and self-regulated learning before and after the intervention in the two groups

**Indicators**	**Self-directed learning**						**Self-regulated learning**	
	**Self-control**		**Self-engagement**		**Self-management**		** M(IQR)**	
	** M(IQR)**		** M(IQR)**		** M(IQR)**			
**Groups**	**TBL**	**Web Quest**	**TBL**	**Web Quest**	**TBL**	**Web Quest**	**TBL**	**Web Quest**
**Before **	50(5)	46(2.25)	57(4.50)	57(11)	54(4)	46(6.75)	51(6)	52(7)
**After**	52(2)	53(4)	57(5)	57(7)	45(5)	50(6)	47(11)	53(7)

Mean (IQR): Median and inter quartile range

The comparison of the students’ mean scores in the two groups using the Mann–Whitney U test showed that although at the beginning of the experiment, both groups had similar scores, by the end of the experiment the participants’ self-directed learning indices (self-management and self-regulated learning) were different (p=0.04 and 0.01, respectively). There was no significant difference between the pre- and post-test scores in total self-directed learning for both groups. Self-control in the pre-test was significant, but there was no significant difference after the intervention. This means that educational intervention had no effect on the self-control levels of both groups (Table 2).

**Table 2:** Mean difference of self-directed learning and self-regulated learning between the groups before and after the intervention

**Indicators **	**Self-directed learning** **Mean of rank**																	
	**Self-control **					**Self-engagement**					**Self-management**				**Total self-directed learning **			
**Groups**	**Web Quest**	**TBL**	**U Mann-Whitney**	**p**		**Web Quest**	**TBL**	**U Mann-Whitney**	**p**		**Web Quest**	**TBL**	**U Mann-Whitney**	**p**	**Web Quest**	**TBL**	**U Mann-Whitney**	**p**
**Before**	41.53	29.12	2.56	0.01		37.06	33	0.84	0.39		33.81	36.16	0.49	0.62	34.29	36.71	0.51	0.60
**After**	35.72	37.33	0.64	0.73		38.33	34.57	0.76	0.44		40.75	31.11	3.94	0.04	3.93	43.24	2.49	0.01

Analysis was fromU Mann-Whitney Test for comparing within group

As shown in Table 3, the results of the Wilcoxon test demonstrated that self-management learning and self-control had changed in the case of the web-based team learning approach (p=0.006 and p=0.001, respectively). With the TBL approach, however, the values of the indices of self-control and self-management were significant (p=0.001 and p=0. 001, respectively). This means that these indices changed after the intervention. Overall, it seems that both approaches had an effect on the self-control and self-management skills of students.

**Table 3:** Mean scores of educational indexes within groups

**Groups**	**Web Quest method**			**Team based learning **		
**Indexes**	**Mean of rank **	**z**	**p**	**Mean of rank**	**z**	**p**
**Self-control**	24.22±18.04	2.72	0.006	9.58±18.65	3.99	0.001
**Self-engagement**	17.42±15.33	9.28	0.35	17.65±15.20	0.67	0.49
**Self-management **	18±11.14	3.48	0.001	18.87±3.33	4.92	0.001
**Total self directed learning**	18.35±3.14	1.25	0.21	21.94±12.50	1.67	0.09
**Self-regulated** **learning**	19.41±11.04	1.60	0.10	21.55±15.02	1.60	0.10

Analysis from Wilcoxon test

The other results showed that the scores related to learning and academic success were higher in the integrated-learning group than in the team-based group (Table 4).

**Table 4:** The groups' final exam mean scores (out of 100)

**Method of teaching **	**Mean± SD**	**T**	**p**
**WebQuest (N=38)**	67.08±6.43	3.33	0.002
**Team-based learning (N=39)**	59.08±6.43		

Analysis from student t- test

With regard to the students’ satisfaction with the teaching approaches, 65% were satisfied from a very strong to a strong degree, 20% were satisfied from a moderate to a good degree, while the rest were satisfied from a poor to a very poor degree. In their written answers to open question, the students reported that the approaches were effective in providing in-depth learning, real experience, non-passivity and student-centeredness, teachers caring about and devoting their energy on the students, and good class atmosphere and flexibility. The approaches also motivated the students, raised their interest in the lessons, were compatible with their learning styles, and were modern and attractive compared with conventional teaching methods. 

## Discussion

The objective of this study was to compare the effects of cooperative learning methods using WebQuest and TBL as teaching strategies on the students’ self-direction, self-regulation, and academic achievement. 

The results showed that WebQuest can change the students’ self-control and self-management in learning as well as its direction. This method also increases the students’ self-regulation. Some evidence emphasizes the effects of information and communication technology on increasing the students’ higher thinking and self-direction (29). The effects of the WebQuest approach and the use of Internet technology on self-direction sub-categories have been confirmed by other studies, confirming the findings of the present study. In their study, Watts and Lloyd (2004) asked the students to use information technology to do some assignments; the students actively participated in the discussions and completed the tasks very quickly and in a self-directed manner (30). In another study, the learners reported that E-learning increased their responsibility and freedom in learning, and their active participation in controlling the learning processes helped them use resources and strategies more efficiently (31).This result confirms the effect of WebQuest on the students’ self-direction as well as on increasing their self-regulated learning; this corresponds with the results of the present study.

Our results showed that WebQuest affected the students’ learning more than TBL did. Similarly, many researchers have confirmed the contribution of WebQuest to learning and academic success. Kulik and Kulik (1991) showed that computer-based approaches improve learning; the grades of the students in the intervention group increased by 20% compared with those of the students who did not use computers (32). In Hasanian’s study (2006) entitled “Using WebQuest and Technology to Support Learning”, most students regarded WebQuest as an incentive to achieving academic success. The researcher concludes that WebQuest is an exciting tool and certainly encourages interactional learning (33). In their comparative study of the effects of WebQuest and lecturing on the students’ ECG interpretation skills, Najafi et al. (2013) concluded that the students subject to Web Quest were more successful (34). Similarly, in their study, Baghaei et al. (2012) reported that Web-based learning improved the learning levels of nursing students (35). Tuan (2011) also used WebQuest to teach reading, which led to an improvement in the learners’ reading skills (36).

The present study showed that WebQuest was an effective strategy for increasing the students’ self-control and self-management skills. This may be due to the use of problem-solving strategies in this approach. In the present study, case-based learning was used to design a WebQuest strategy as a learning plan and to shape the team-based activities.  

Moon SookYoo’s study in Korea (2010) studied the effects of case-based learning (CBL) on the learning motivation of 44 junior nursing students. The results showed a significant increase in the learning motivation of the students in the CBL group, which indicates that by encouraging self-directed learning and raising interest, CBL motivates learning (37). By introducing real cases into medical education, CBL increases the students’ interest, makes effective learning possible, and gives students an overview of the disorders studied rather than separate descriptions of the diseases of each organ.

In our study, TBL affected the students’ self-control and self-management. This approach is an essential technique in cooperative learning, and its effect on the students’ learning as well as other advantages has been confirmed by many studies. Searle et al. (2003) showed that TBL encourages studying outside the classroom as well as teamwork and participation within the class (38). Another study shows that preparation outside the class is one of the components of TBL, which encourages the students to study regularly and prepare before going to class, and consequently creates a good atmosphere for self-directed and active learning (39, 40). It is clear that the employment of either strategy results in positive outcomes, and students with different learning abilities and methods will benefit from a blend of these approaches. Analysis of the findings also revealed a decrease in the students’ self-directed learning using the team-based approach.

According to Thompson et al. (2007), the teacher, organization/curriculum, students, and features of the lessons are important factors in TBL and need to be considered before a TBL lesson plan is developed (17). For instance, pre-class preparation may not be attractive to or possible for all learners; the brevity of the course may prevent the maturation of the team; and the teachers must carefully design the readiness assurance tests and prepare the learners for them (41). In addition, students who lack the skills for self-directed learning will experience anxiety and frustration with both educational programs (42); Iranian students are not trained for life-long learning before they enter the university; hence, they may be unable to adapt to the freedom and independence that characterize the lesson plans and class management of these programs. These students may be affected by the loss of the teacher as the leader and determiner of their activities (43). Therefore, it is essential to train the students in self-directed learning through workshops and seminars before applying these approaches.

Other results show that the WebQuest group sees an increase in its students’ self-regulation median range. Self-regulation is a primary variable that enables successful learners to manage their cognitive, metacognitive, and motivational learning (44). In fact, learners control their behavior and facilitate learning by the motivations that they create themselves. They learn how to evaluate their knowledge before an exam and make the necessary adjustments to their learning strategies to increase their chances of success (45).

TBL, on the other hand, is not directly associated with better self-regulation and self-engagement; however, some researchers and students of various levels of knowledge come together as a team. Those who are well-prepared perform well on the individual readiness assurance tests and distinguish themselves. The less ready students cannot contribute to their group’s knowledge and critical thinking and are consequently motivated to be more prepared the next time to perform better on the individual readiness assurance tests and meet their teammates’ expectations (46-49). Thus, the students will have better metacognitive self-regulation.

Recent research has also found that TBL has positive effects on the students’ self-control and self-management. According to Michaelsen et al. (1997), group answering in TBL leads to students’ higher efficiency and better management of available resources (50): self-regulated learners will effectively use their teammates as sources of knowledge. Similarly, Zimmerman and Schunk (2007) pointed to the key role of resource-management cognitive strategies in self-regulated learning (51). It was believed that in integrated learning, where TBL was accompanied by the WebQuest method and learners were allowed more freedom, they felt the need for more self-regulation, and accordingly tried to achieve more independence and self-regulation; however, the expectation was not realized.

Studies show that self-regulated learning significantly improves the learners’ academic success; however, despite the higher self-regulation in the TBL approach in this study, the learners in the integrated learning group achieved higher learning and academic success scores. Therefore, it can be concluded that the synergy achieved in integrated learning has greater effects than self-regulation. Similarly, in Nieder et al.’s study of medical students’ success in anatomy and embryology, the mean scores of the students trained through TBL were no different from those of the students in the previous year who were not taught using TBL (52). In a study at the medical college of Boonshoft, two groups of students were trained separately using CBL and TBL; the results did not show a significant difference between the performances of the two teams on the comprehensive exams of CCE (53).

Overall, a review of the studies’ results reveals that a combination of different teaching and learning approaches involving students in searching for resources will result in improved learning. In integrated learning, where students utilize a broad range of online resources, learning becomes personalized. Students will use the learning opportunities based on their individual qualities, and the greater flexibility of the learning environment will increase the students’ satisfaction. This point is elaborated in the present study and in the qualitative analysis.

The students in this study reported that the WebQuest strategy contributed to their in-depth learning; offered real experiences, student-centeredness, teachers’ devotion, better class atmosphere and flexibility; encouraged their greater interest in the subjects’ motivation; were compatible with their learning; and were novel and attractive. Similarly, in their study of students in a cardiac course (2010), Conway et al. replaced eight hours of lecturing with self-directed learning, and the case study discussions were conducted based on TBL to increase the interaction among the students. The results showed an increase in the teachers’ and students’ satisfaction with both changes (54). These results are in agreement with those of the present study (55-58).

The ever-increasing efforts toward involving learners in their education aim at better preparing them for performance in today’s world. The employment of integrated methods (WebQuest in team-based and case-based learning) will positively influence the students’ self-directed learning and academic success. Therefore, it is hoped that these approaches will be used more extensively in the future.

One limitation of this study was the small sample size. The participants of this study consisted of 77 undergraduate nurses, and this may not fully allow the findings to be generalized for the whole student population. Moreover, this study was experimental and comparative in nature, with traditional and other active methods of teaching, such as problem-based learning. Despite these limitations, this research is significant due to the scarcity of studies related to CBL, TBL, and WebQuest as an integrated method; it is also compared with the team-based method as one known to many. Strength of this study was the combination of qualitative and quantitative methods employed. Further studies are needed to confirm the effects of integration in nursing education. 

## Conclusion

Our students need to be prepared to face various professional challenges. By employing modern educational approaches and studying their effects on the students’ academic success and cognitive skills, we can ensure a suitable combination of approaches and find ways to use them more efficiently. This will help the students become more successful in their studies and acquire the necessary professional skills for future performance.
